# Use of digital health technologies in periprocedural pediatric cardiac ablation

**DOI:** 10.1016/j.cvdhj.2024.03.004

**Published:** 2024-04-08

**Authors:** Nathan Miller, David Catherall, Anthony G. Pompa, Lisa Roelle, Tracy Conner, William B. Orr, Jennifer N. Avari Silva

**Affiliations:** ∗ Electrophysiology Laboratory, St. Louis Children’s Hospital, St. Louis, Missouri; † Washington University School of Medicine, St. Louis, Missouri; ‡ Division of Pediatric Cardiology, Washington University in St. Louis, St. Louis, Missouri; § Department of Biomedical Engineering, Washington University in St. Louis, St. Louis, Missouri

**Keywords:** Pediatrics, Catheter ablation, Ablation, Kardia, KardiaMobile, Digital health, Telehealth, Telemedicine, Electrophysiology study, Electrocardiography, ECG

**Key Findings**

• Patients scheduled for electrophysiology (EP) study and possible catheter ablation undergoing a preprocedure telehealth visit with the EP provider can provide the patient and family increased satisfaction and overall knowledge regarding their procedure.
• Patients were overall satisfied with the use of the KardiaMobile 6L (AliveCor Inc, Mountain View, CA) device and application.
• EP physicians were satisfied with the quality of the KardiaMobile 6L electrocardiogram (ECG) data.
• Patients who exhibit subtle pre-excitation may require a standard 12-lead ECG tracing postablation to access for recurrence.

## Introduction

Advances in digital health technologies can democratize health care access, improving communication between patients and providers.^1^^,^^2^ New technologies offer opportunities to redesign patient care models.^3^ Telehealth (TH) use in pediatric cardiology patients has shown feasibility and effectiveness in expanding access, expediting care, and reducing costs.^2^, ^3^, ^4^, ^5^ With the increase in direct-to-consumer heart rate and rhythm remote monitoring, electrophysiology (EP) is well suited to digital health technology initiatives.^4^^,^^6^ The KardiaMobile® 6L (AliveCor Inc, Mountain View, CA) (KM 6L) has shown to provide comparable data to a standard 12-lead electrocardiogram (ECG).^7^, ^8^, ^9^, ^10^

We aim to investigate patient/family satisfaction with preprocedure TH visits, the use of KM 6L device during postablation follow-up, and the EP providers’ satisfaction with the quality of KM 6L ECG.

## Methods

Approval was obtained from the Washington University School of Medicine Institutional Review Board. Inclusion criteria included patients undergoing an EP study with a smartphone (Apple or Android). Patients were excluded if they were non–English speaking, lacked access to the patient portal (MyChart), lacked access to a smartphone, or had EP study without ablation.

Data collected from the electronic medical record included demographic data, participation in a preprocedure TH appointment, and procedural information. Written consent, and assent as appropriate, were obtained. Participants were provided with a KM 6L and study documents, including tip sheets on recording and sending the KM ECG tracing via the patient portal, a preprocedural TH survey, a KM 6L device usability survey, and a prepaid shipping label with envelope to return items.

Participants were contacted prior to follow-up appointment to transmit the KM 6L tracing, complete the surveys, and return items in the provided envelope. After the postprocedure TH visit, the EP physician completed a survey regarding the quality of the KM 6L tracing. Survey responses used a 5-point Likert scale, ranging from 1 (strongly disagree) to 5 (strongly agree).

### Statistical analysis

Descriptive data are presented as a mean ± range with standard deviation or percentages.

## Results

### Patient demographic data

Twenty-eight patients were enrolled; 75% (21/28) were male. Average patient age was 13 ± 4 years (range 2–18 years). Average weight was 63.1 ± 26.3 kg and average height was 161.4 ± 21.8 cm. EP diagnoses included atrioventricular nodal reentrant tachycardia (AVNRT, 10/28, 36%), Wolff-Parkinson-White (WPW, 9/28, 32%), and concealed accessory pathway–mediated tachycardia (9/28, 32%) (Table 1).

**Table 1:** Demographic data

Feature	Result (n = 28 patients)
Sex	
Female	7 (25%)
Male	21 (75%)
Age (years)	13.2 ± 4.0
Weight (kg)	63.1 ± 26.3
Height (cm)	161.4 ± 21.8
Preprocedural telehealth visit	14 (50%)
Diagnosis at time of EPS†	
Atrioventricular nodal reentrant tachycardia	10 (36%)
Manifest accessory pathway	9 (32%)
Concealed accessory pathway	9 (32%)
Acute procedural success	26 (92.3%)
KardiaMobile monitor 6L ECG tracing received postablation	24 (85.7%)
KardiaMobile monitor 6L returned	27 (96.4%)

ECG = electrocardiogram; EPS = electrophysiology study.

† Diagnosis represents the findings during the electrophysiology study.

### Preprocedure TH data

**Figure 1:**
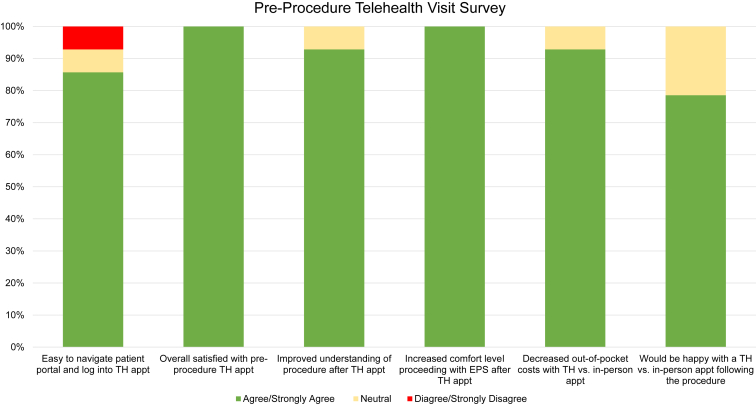
Results from the patient preprocedure telehealth (TH) visit survey. Statements are shown along the x-axis and percentage response on the y-axis. The green bars represent percentage of patients/families that strongly agreed/agreed with the statement. The yellow bars represent the percentage of patients who had a neutral response and red bars represent those responses that strongly disagreed/disagreed.

Fifty percent (14/28) of patients had a preprocedure TH appointment (Table 1) with surveys demonstrating 12 patients (12/14, 86%) reporting it was easy to navigate the patient portal and log in to their TH appointment. All 14 patients (100%) were satisfied with their TH appointment and higher level of comfort moving forward with the EP study. A total of 93% of patients (13/14) reported a better level of understanding of the EP procedure post visit, and 79% of patients (11/14) reported that they would be happy with a postprocedure TH appointment (Figure 1).

### Postprocedure KM 6L data

Twenty-four patients (24/28, 85.7%) obtained a KM 6L tracing, sent the tracing in, and completed the survey. Four patients (4/28, 14.3%) failed to send their tracing either because (1) they decided not to (n = 2), (2) they had issues using their smartphone (n = 1), or (3) the patient was unreachable following the procedure (n = 1). Twenty-seven patients (27/28, 96.4%) returned the KM 6L device.

**Figure 2:**
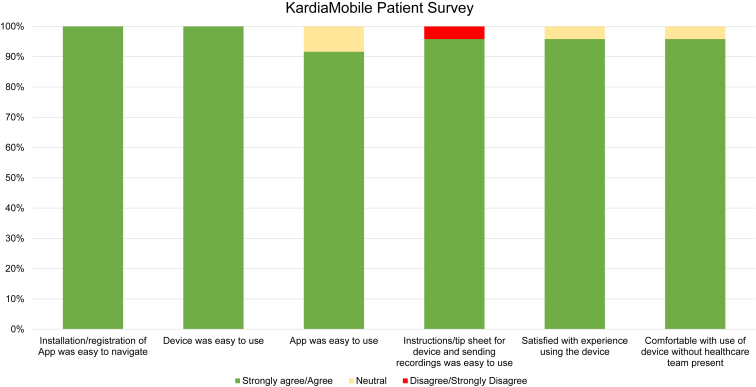
Results from the KardiaMobile patient survey. Statements are shown along the x-axis and percentage response on the y-axis. The green bars represent percentage of patients/families that strongly agreed/agreed with the statement. The yellow bars represent the percentage of patients who had a neutral response and red bars represent those responses that strongly disagreed/disagreed.

All respondents on the postprocedure KM 6L survey reported that the KM application was easy to navigate and that the device was easy to use. Most notably, 92% (22/24) reported that the application was easy to use, 96% (23/24) felt that the instructions and tip sheet for device use and sending recordings were easy to use, 96% (23/24) were satisfied with the experience of using the device, and 96% (23/24) felt comfortable with using the device without the health care team present (Figure 2).

EP providers felt that the KM 6L ECG provided a high-quality, clinically usable ECG with sufficient data to assess postablation ECGs, and that the KM 6L ECG in post–EP procedure follow-up is an acceptable alternative in place of an in-person 12-lead ECG. However, 2 providers commented that the device would be less suitable for detecting subtle pre-excitation and that a follow-up 12-lead ECG may still be needed in these cases.

## Discussion

This study demonstrates the feasibility and satisfaction of implementing (1) pre–EP procedure TH visits and (2) postablation ECG with a KM 6L in pediatric patients. Patients and families found the KM 6L device and application easy to use, reporting overall satisfaction. Physicians reported comfort using the KM 6L in certain postablation patients.

Participants and families who had a preprocedure TH visit reported better understanding and having a higher level of comfort moving forward with the EP study, which aligns with previous studies.^2^^,^^4^^,^^5^^,^^10^ This is particularly important for pediatric EP procedures, which are typically performed at referral centers and for which patients often have not met with the proceduralist prior to the day of the procedure.

The KM 6L device allows anyone the ability to obtain 6L ECGs outside a hospital setting. The patient/family survey results revealed overall satisfaction with the use of the device and smartphone application. This is consistent with previous studies, which also reported overall satisfaction and ease of use when using a KM device in the outpatient setting.^9^^,^^11^ Patient/family satisfaction may directly relate to physician review of the 6L ECG in the patient portal, which allowed for patients to use TH for postprocedure follow-up.

**Figure 3:**
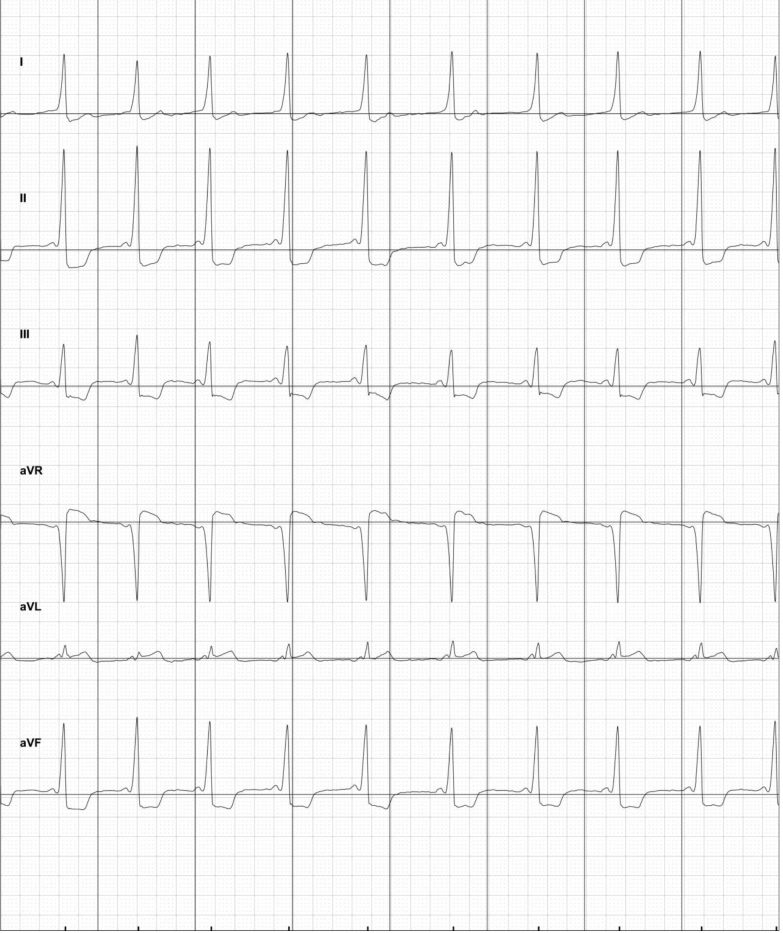
KardiaMobile 6L (AliveCor Inc, Mountain View, CA) electrocardiogram tracing in patient with recurrence of pre-excitation at time of postablation follow-up.

In our study, most enrolled patients underwent ablation for AVNRT or an accessory pathway. Physicians felt that the 6L ECG was a reasonable tool to assess rhythm postablation for those with AVNRT. In patients who had recurrent, overt pre-excitation, the 6L ECG successfully detected recurrence in 1 patient (Figure 3). However, clinicians had reservations about the ability to identify subtle pre-excitation with the 6L KM. Physicians commented on their surveys that subtle pre-excitation preprocedure was difficult to see on standard 12-lead ECG for 2 patients and that the 6L KM may not be ideal to assess for recurrent pre-excitation. This cohort of patients may not be well suited to use of 6L KM tracings postprocedure, though this has not been systematically studied.

### Study limitations

This study is limited by a small patient population, a nonrandomized single-center investigation, and the need for additional data to compare outcomes to the standard of care. Reliance on family access to smartphones, internet reliability, and technological literacy are also potential limitations. Given possible failure to return the device, larger-scale implementation may require a technology contract to mitigate the risk of lost equipment. Finally, evolving regulatory and reimbursement landscape around telemedicine may impact the future of TH economics. During this study, Medicare changed its reimbursement policies, causing our institution to no longer allow new patient TH appointments.

## Conclusion

Preprocedure TH visits for pediatric patients undergoing EP study and catheter ablation can aid in increased satisfaction and understanding of the procedure. Postablation, patients/families were satisfied with the ease of use of the KM 6L and EP physicians were pleased with the quality of data in certain patient populations. Patients with subtle pre-excitation may require 12-lead ECG tracings postablation to assess for recurrence.

## Disclosures

JNAS has the following disclosures: Kardia 6 lead devices supplied by AliveCor to our group to carry out the study. No other disclosures.

## References

[1] Cremades M., Ferret G., Parés D. (2020). Telemedicine to follow patients in a general surgery department. A randomized controlled trial. Am J Surg.

[2] Kruse C.S., Krowski N., Rodriguez B., Tran L., Vela J., Brooks M. (2017). Telehealth and patient satisfaction: a systematic review and narrative analysis. BMJ Open.

[3] Mugnai G., Volpiana A., Cavedon S., Paolini C., Perrone C., Bilato C. (2021). Boosting telemedicine through remote monitoring of cardiac electronic devices during the Italian COVID-19 outbreak. Cardiol J.

[4] Preminger T.J. (2022). Telemedicine in pediatric cardiology: pros and cons. Curr Opin Pediatr.

[5] Schweber J., Roelle L., Ocasio J. (2022). Implementation and early experience of a pediatric electrophysiology telehealth program. Cardiovasc Digit Health J.

[6] Lu J.K., Sijm M., Janssens G.E., Goh J., Maier A.B. (2023). Remote monitoring technologies for measuring cardiovascular functions in community-dwelling adults: a systematic review. Geroscience.

[7] Azram M., Ahmed N., Leese L. (2021). Clinical validation and evaluation of a novel six-lead handheld electrocardiogram recorder compared to the 12-lead electrocardiogram in unselected cardiology patients (EVALECG Cardio). Eur Heart J Digit Health.

[8] Orchard J.J., Orchard J.W., Raju H., La Gerche A., Puranik R., Semsarian C. (2021). Comparison between a 6-lead smartphone ECG and 12-lead ECG in athletes. J Electrocardiol.

[9] Gropler M.R.F., Dalal A.S., Van Hare G.F., Silva J.N.A. (2018). (2018) Can smartphone wireless ECGs be used to accurately assess ECG intervals in pediatrics? A comparison of mobile health monitoring to standard 12-lead ECG. PLoS One.

[10] Kleiman R., Darpo B., Brown R. (2021). Comparison of electrocardiograms (ECG) waveforms and centralized ECG measurements between a simple 6-lead mobile ECG device and a standard 12-lead ECG. Ann Noninvasive Electrocardiol.

[11] Roelle L., Ocasio J., Littell L. (2022). Expanding telehealth through technology: use of digital health technologies during pediatric electrophysiology telehealth visits. Cardiovasc Digit Health J.

